# Associations between multimorbidity and depression among breast cancer survivors within the UK Biobank cohort: a cross-sectional study

**DOI:** 10.1186/s12885-021-08409-z

**Published:** 2021-05-31

**Authors:** Murray Foster, Claire L. Niedzwiedz

**Affiliations:** 1 College of Medical, Veterinary and Life Sciences, University of Glasgow, Glasgow, UK; 2 College of Medical, Veterinary and Life Sciences, Institute of Health & Wellbeing, University of Glasgow, Glasgow, UK

**Keywords:** Breast cancer, Multimorbidity, Comorbidity, Depression, Epidemiology, Survivorship, Mental health

## Abstract

**Background:**

Advances in the early detection of cancer and its treatment have resulted in an increasing number of people living with and beyond breast cancer. Multimorbidity is also becoming more common in this population as more people live longer with breast cancer and experience late effects of cancer treatment. Breast cancer survivors have heightened risk of depression, but to what extent multimorbidity affects the mental health of this population is less clear. This study aims to investigate the association between multimorbidity and depression among women living with and beyond breast cancer in the UK Biobank cohort.

**Methods:**

Data from UK Biobank (recruitment during 2006 to 2010, aged 40–70 years) were used to identify 8438 women with a previous diagnosis of breast cancer via linked cancer registries in England, Scotland and Wales. The lifetime number of chronic conditions was self-reported and multimorbidity defined as 0, 1, 2, 3, 4 or 5+. The Patient Health Questionnaire (PHQ-2) was used to define participants that were likely to have depression based on their symptom reporting at baseline. Logistic regression models were used to analyse the associations between multimorbidity and depression, accounting for a number of potential sociodemographic confounding variables (including age, ethnicity, socioeconomic deprivation, education level and marital status) and characteristics related to the cancer (number of years since diagnosis and recurrence/secondary cancer).

**Results:**

Multimorbidity was common among breast cancer survivors, with 32.9% of women experiencing one and 30.1% experiencing two or more chronic health conditions. Hypertension (25.8%), painful conditions (18.3%), and asthma (11.6%) were the three most common co-morbid conditions. 5.3% of participants had current depression. A strong, dose-response relationship was found between multimorbidity and the likelihood of depression (OR = 2.09, 95% CI: 1.56–2.79 for two conditions and OR = 6.06, 95% CI: 3.63–10.14 for five or more conditions).

**Conclusions:**

Multimorbidity and depression were strongly associated among female UK Biobank participants with a previous breast cancer diagnosis. This association became increasingly pronounced as the number of chronic comorbid conditions increased. As more people survive cancer for longer, increasing recognition and support for multimorbidity and its impact on mental health is needed.

**Supplementary Information:**

The online version contains supplementary material available at 10.1186/s12885-021-08409-z.

## Background

Breast cancer is one of the most frequently diagnosed cancers around the world, representing 11.6% of all cancer cases [1]. It is the most common type of cancer in the United Kingdom, where it makes up 15% of all new cancer cases and 7% of all deaths due to cancer [2]. Advancements in medical care have led to substantial improvements in breast cancer survival over the past 40 years, achieved though ever improving treatment, supportive care, screening and earlier diagnosis [3]. With 85% of women now surviving breast cancer for 5 years or more in the UK, a deeper understanding of the psychological impact of the disease is becoming increasingly important and a growing clinical priority [2, 4].

Multimorbidity, defined as the presence of two or more chronic health conditions [5], is an additional aspect of medicine progressively requiring further study, due to the rising population of older people. 27% of primary care patients have multimorbidity [6], a figure greatly increased in individuals with cancer, where 69–88% of the population report at least one comorbid disease [7]. The increased prevalence of multimorbidity among people with cancer can be partially explained by adverse effects of cancer treatment, and shared etiological factors for cancer and comorbid conditions [8]. Women diagnosed with breast cancer have been shown to have a higher risk of developing new comorbidities when compared to women without a history of cancer [9]. Multimorbidity among people with cancer is therefore an increasing concern which likely has implications for mental health, but research into multimorbidity, and its effects on mental health, among this population remains limited [10].

Both breast cancer and multimorbidity are factors associated with an increased risk of depression [11]. Depression is two to three times more likely in people with multimorbidity compared to those without [12]. A 2018 meta-analysis found strong evidence that breast cancer survivors have an increased risk of depression compared to women with no prior cancer [13] and a Scottish study found the prevalence of depression in breast cancer patients to be 9% [14]. A recent study using the UK Clinical Practice Research Datalink (CPRD) found that women with breast cancer had 1.3 times higher risk of depression and anxiety compared to matched controls, which decreased over time but remained elevated for several years after breast cancer diagnosis [15]. With breast cancer patients having a heightened likelihood of multimorbidity, the combined impact of both of these two factors upon depression has clear importance as an avenue of research.

The individual links between multimorbidity, depression, and breast cancer have been relatively well researched, but the interplay between the combination of the three has a much more limited representation in the current literature [16]. This study therefore aims to investigate the association between multimorbidity and depression among a UK cohort of breast cancer survivors.

## Methods

### Study population

UK Biobank contains data from over 500,000 volunteers, recruited in the period between 2006 and 2010 from across England, Scotland, and Wales. The volunteers were aged between 40 and 70 at the time of recruitment, and provided data from touchscreen questionnaires, physical measurements, genotyping and longitudinal follow-up, with further data continuing to be added [17]. The data are linked to cancer registries, allowing identification of individuals previously diagnosed with breast cancer prior to baseline recruitment.

Included in this study were women with a primary diagnosis of a malignant neoplasm of the breast (International Classification of Diseases 10th Revision (ICD-10) code C50 and ICD-9 code 174). 9364 women matching this criterion were identified.

UK Biobank received ethical approval from the NHS National Research Ethics Service North West (16/NW/0274) and was performed in accordance with the Declaration of Helsinki. Informed consent was obtained for all UK Biobank participants. The analyses in this study were performed under UK Biobank application number 41686.

### Outcome variable

Current depression was the primary outcome variable, which was assessed using the validated two-item Patient Health Questionnaire (PHQ-2) at baseline [18]. The PHQ-2 operates by asking participants about two key depressive symptoms (low mood and anhedonia) via two simple questions – in the 2 weeks prior how often have they “felt down, depressed, or hopeless” (data field 2050), and how often they “had little interest or pleasure in doing things” (data field 2060). Responses available to participants were “not at all” (assigned a score of 0), “several days” (assigned a score of 1), “more than half the days” (2), and “nearly every day” (3). Participants answering “do not know” or “prefer not to answer” were coded as missing. The combined total provides a score from 0 to 6, with participants scoring at least three categorized as having probable current depression and those scoring less than three categorized as no current depression [18]. We chose to use a measure of current depression to limit the possibility of reverse causation as compared with using a measure of lifetime depression (reported by 7.2% of the sample). We selected the cut-off value of ≥3 as a measure of more severe depression. Using this approach, 5.3% of the sample were classed as currently depressed, compared to 16.6% using a cut-off value of ≥2 [19]. The proportion of people classed as currently depressed using the higher cut-off threshold is also more similar to the measure of self-reported lifetime depression.

### Exposure variable

Multimorbidity was measured by a count of the number of self-reported doctor diagnosed lifetime chronic health conditions recorded during the baseline nurse-led interview (data fields 20001 and 20002) and informed by previous literature on multimorbidity [5, 20, 21]. The physical and mental health conditions reported by participants were classified into 43 groups following a previously published approach [20–22]. We excluded lifetime depression, cancer and prostate disorder (as our sample was exclusively women) from the count of co-morbid conditions and multimorbidity was coded as 0, 1, 2, 3, 4 or 5+. Please refer to Additional file 1: Table S1 for full details of the chronic health conditions considered.

### Potential confounding variables

We included a number of potential confounding variables: age (5-year bands), education level (degree, non-degree level), socioeconomic deprivation (measured using the Townsend Index [23] and divided into quartiles), ethnicity (white, non-white), marital/cohabitation status (living with spouse/partner, not living with spouse/partner), whether the participant had been previously diagnosed with cancer more than once (yes, no), and time since the primary breast cancer diagnosis (less than 1 year, 1–3 years, 3–5 years, 5+ years).

### Statistical analysis

Descriptive statistics were used to summarise the sample and the most commonly reported chronic health conditions. Logistic regression models were calculated to analyse the association between multimorbidity and the odds of depression. Model 1 included the number of chronic conditions and patient age as predictors. Model 2 additionally included ethnicity, education level, socioeconomic deprivation, marital/cohabitation status, more than one cancer diagnosis and number of years since the primary breast cancer diagnosis as predictors. These models were used to calculate odds ratios with 95% confidence intervals and predicted probabilities (using Stata’s margins command with the atmeans option) to determine the effect sizes. A significance level of *p* < 0.05 was used in this study. Missing data were excluded (*n* = 926, 9.9%) from the analysis, with most missing data being found for PHQ-2 (*N* = 675). A small amount of missing data was also observed for multimorbidity (*N* = 66), ethnicity (*N* = 34), education level (*N* = 168), marital/cohabitation status (*N* = 51), and socioeconomic deprivation (*N* = 12). After the exclusion of missing data 90.1% of participants were included in the final analysis sample (*N* = 8438). Analyses were completed in R version 4.0.2 and Stata/MP 16.0.

## Results

63% of participants had at least one other chronic condition, with 30.1% having two or more co-morbid conditions (Table 1). Hypertension affected the highest proportion of participants (25.8%) (Table 2 displays the other most prevalent conditions). 5.3% of the study population were defined as having current depression as defined by the PHQ-2. 27.8% had been diagnosed with cancer more than once, which could include a recurrence of breast cancer or a secondary cancer. The majority of participants had been survivors of breast cancer for more than 5 years (63.4%), with 5.6% diagnosed in the past year. Depression was most common among people with co-morbid irritable bowel syndrome, diabetes, and migraine, with 11.9, 11.6, and 10.1% of participants reporting the two conditions, respectively (Table 2).

**Table 1:** Description of the sample

	Current depression (PHQ-2 ≥ 3) N (%)	No current depression (PHQ-2 < 3) N (%)	Total sample N (%)
**Age group**			
40–44	19 (4.2%)	198 (2.5%)	217 (2.6%)
45–49	49 (10.9%)	480 (6.0%)	529 (6.3%)
50–54	79 (17.6%)	935 (11.7%)	1014 (12.0%)
55–59	97 (21.6%)	1501 (18.8%)	1598 (18.9%)
60–64	124 (27.6%)	2658 (33.3%)	2782 (33.0%)
65–70	81 (18.0%)	2217 (27.7%)	2298 (27.2%)
**Deprivation Quartile**			
1 (most advantaged)	85 (18.9%)	2132 (26.7%)	2217 (26.3%)
2	93 (20.7%)	2120 (26.5%)	2213 (26.2%)
3	99 (22.1%)	2007 (25.1%)	2106 (25.0%)
4 (least advantaged)	172 (38.3%)	1730 (21.7%)	1902 (22.5%)
**Education Level**			
Degree	112 (24.9%)	2413 (30.2%)	2525 (29.9%)
Non-degree	337 (75.1%)	5576 (69.8%)	5913 (70.1%)
**Ethnicity**			
White	410 (91.3%)	7799 (97.6%)	8209 (97.3%)
Non-white	39 (8.7%)	190 (2.4%)	229 (2.7%)
**Marital/cohabitation status**			
Living with spouse/partner	256 (57.0%)	5491 (68.7%)	5747 (68.1%)
Not living with spouse/partner	193 (43.0%)	2498 (31.3%)	2961 (31.9%)
**Diagnosed with cancer more than once**			
No	328 (73.0%)	5761 (72.1%)	6089 (72.2%)
Yes	121 (27.0%)	2228 (27.9%)	2349 (27.8%)
**Number of years since original diagnosis of breast cancer**			
Less than 1 year	35 (7.8%)	434 (5.4%)	469 (5.6%)
1–3 years	85 (18.9%)	1374 (17.2%)	1459 (17.3%)
3–5 years	68 (15.1%)	1093 (13.7%)	1161 (13.8%)
5+ years	261 (58.1%)	5088 (63.7%)	5349 (63.4%)
**Number of chronic health conditions**			
0	115 (25.6%)	3006 (37.6%)	3121 (37.0%)
1	128 (28.5%)	2651 (33.2%)	2779 (32.9%)
2	96 (21.4%)	1427 (17.9%)	1523 (18.1%)
3	54 (12.0%)	584 (7.3%)	638 (7.6%)
4	34 (7.6%)	204 (2.6%)	238 (2.8%)
5+	22 (4.9%)	117 (1.5%)	139 (1.6%)
**N**	449 (5.3%)	7989 (94.7%)	8438 (100%)

**Table 2:** The 10 most common co-morbidities among 8438 women with breast cancer in UK Biobank and the proportion of participants with and without current depression

Co-morbid condition	Current depression (PHQ-2 ≥ 3) N (%)	No current depression (PHQ-2 < 3) N (%)	Total sample with co-morbid condition N (%)
**Hypertension**	152 (7.0%)	2021 (93.0%)	2173 (25.8%)
**Painful conditions**	102 (6.6%)	1439 (93.4%)	1541 (18.3%)
**Asthma**	64 (6.5%)	919 (93.5%)	983 (11.6%)
**Thyroid conditions**	56 (6.3%)	833 (93.7%)	889 (10.5%)
**Dyspepsia**	51 (7.6%)	623 (92.4%)	674 (8.0%)
**Osteoporosis**	28 (6.7%)	390 (93.3%)	418 (5.0%)
**Diabetes**	42 (11.6%)	320 (88.4%)	362 (4.3%)
**Migraine**	28 (10.1%)	249 (89.9%)	277 (3.3%)
**Eczema**	13 (5.9%)	206 (94.1%)	219 (2.6%)
**Irritable bowel syndrome**	25 (11.9%)	185 (88.1%)	210 (2.5%)

Women with one co-morbid chronic condition had higher odds of experiencing depression, compared to those with no such condition (OR = 1.47, 95% C1:1.13–1.91) in Model 1 which adjusted for age), corresponding to a predicted probability for depression of 4.3% compared to a predicted probability of 2.9% in participants without any comorbidity (Table 3). Two co-morbid conditions were also related to a heightened odds of depression (OR = 2.29, 95% CI: 1.72–3.05). With an increasing number of chronic conditions, the likelihood of depression was increased (OR = 7.10, 95% CI: 4.28–11.76 in participants with 5 or more chronic conditions, corresponding to a predicted probability of depression of 17.7%).

**Table 3:** Results from logistic regression models for the association between the number of chronic health conditions and current depression among 8438 women with breast cancer in UK Biobank

	Model 1				Model 2			
	Odds Ratio	95% Confidence Interval	***P***-value	Predicted probability	Odds Ratio	95% Confidence Interval	P-value	Predicted probability
0 (reference)	1			2.9%	1			2.9%
1	1.47	1.13–1.91	0.004	4.3%	1.42	1.09–1.85	0.009	4.0%
2	2.29	1.72–3.05	< 0.001	6.5%	2.09	1.56–2.79	< 0.001	5.8%
3	3.32	2.35–4.70	< 0.001	9.2%	3.01	2.12–4.27	< 0.001	8.1%
4	6.25	4.10–9.52	< 0.001	15.9%	5.48	3.57–8.42	< 0.001	13.9%
5+	7.10	4.28–11.76	< 0.001	17.7%	6.06	3.63–10.14	< 0.001	15.1%

Model 1: Number of chronic health conditions, age group

Model 2: Number of chronic health conditions, age group, education level, ethnicity, marital/cohabitation status, diagnosed with cancer more than once, number of years since primary breast cancer diagnosis

The associations persisted in Model 2, which adjusted for education level, ethnicity, socioeconomic deprivation, marital/cohabitation status, more than one cancer diagnosis and number of years since the primary breast cancer diagnosis (Table 4). The odds ratio for those with one chronic condition was 1.42 (95% CI: 1.09–1.85) and increased to 6.06 (95% CI: 3.63–10.14) in participants with 5 or more comorbid conditions. This gave a predicted probability of depression of 4.0% in participants with one condition, compared to a probability of 15.1% in participants with 5 or more conditions.

**Table 4:** Results from logistic regression models for the association between the number of chronic health conditions and current depression among 8438 women with breast cancer in UK Biobank (full results from Model 2)

	Odds Ratio	95% Confidence Interval	P-value
**Number of chronic health conditions 0 (Ref = 0)**			
1	1.42	1.09–1.85	0.009
2	2.09	1.56–2.79	< 0.001
3	3.01	2.12–4.27	< 0.001
4	5.48	3.57–8.42	< 0.001
5+	6.06	3.63–10.14	< 0.001
**Age group (Ref = 40–44)**			
45–49	1.06	0.60–1.88	0.834
50–54	0.83	0.49–1.43	0.508
55–59	0.58	0.34–1.00	0.048
60–64	0.40	0.24–0.68	0.001
65–70	0.28	0.16–0.48	< 0.001
**Ethnicity (Ref = white)**			
Non-white	2.90	1.99–4.24	< 0.001
**Education level (Ref = degree level)**			
Non-degree	1.33	1.06–1.67	0.014
**Socioeconomic deprivation quartile (Ref = 1, most advantaged)**			
2	1.04	0.77–1.41	0.813
3	1.05	0.78–1.42	0.752
4 (least advantaged)	1.77	1.33–2.35	< 0.001
**Marital status (Ref = living with spouse/partner)**			
Not living with spouse/partner	1.36	1.11–1.67	0.003
**Diagnosed with cancer more than once (Ref = no)**			
Yes	1.10	0.88–1.38	0.405
**Number of years since original cancer diagnosis (Ref = 1–3 years)**			
Less than 1 year	1.28	0.84–1.95	0.249
3–5	0.95	0.68–1.34	0.787
5+	0.92	0.70–1.20	0.533

## Discussion

Multimorbidity was common among women with breast cancer, with a prevalence which is similar to that found in previous studies of cancer survivors [7]. Our results demonstrated a clear positive association between the presence of multimorbidity and the risk of depression in survivors of breast cancer. This association remained after adjustment for confounding factors and became more pronounced as the number of comorbidities increased. The elevated risk of depression could be related to higher treatment burden, increasing disability and lower quality of life associated with multiple health conditions. Multimorbidity may also cause additional health care cost and financial burden which may adversely affect mental health among cancer survivors [24].

These results are consistent with the small number of published papers investigating this triad of breast cancer, multimorbidity, and depression. Zoorob et al. conducted the study most relevant to our research, studying breast cancer, depression, and multimorbidity among hospitalised women and men in the US [16]. However, this study was limited to an inpatient sample which may not be comparable to the population of people living with breast cancer in the community. Similar to our study, 67.1% of women had a comorbidity, with hypertension the most prevalent. Zoorob found similar increased risk of depression in women with one comorbidity, but our study found a stronger relationship for those with more severe multimorbidity, we found an odds ratio of 5.48 in women with 4 comorbidities, compared to Zoorob’s finding of 2.80 (in women with 4 co-morbidities or greater). Nevertheless, both studies clearly demonstrated a strong association between the number of chronic conditions and the likelihood of depression. Of interest was the finding that most of the chronic comorbidities identified were strongly associated with lifestyle behaviours, which could be partially explained by the high prevalence of physical inactivity in the breast cancer patient population [25].

A 2019 paper authored by Yan et al. performed a similar analysis, but investigated depression and multimorbidity in survivors of all cancer types as opposed to a specific focus on people with breast cancer [26]. They found that cancer survivors with 1–2 chronic comorbidities had greater risk of depression and there was also an additional association between comorbidities and anxiety, with patients with 3 or more comorbidities having 3.39 times the odds of anxiety compared to those without any comorbidity [26]. This study was limited to the population of Shanghai, China. To our knowledge, our study is the first to investigate multimorbidity and depression among people living with breast cancer in the UK and one of the largest studies of breast cancer survivors.

Suppli et al. also investigated the risk for depression after breast cancer in the Danish population, finding comorbidity to be one of the two variables most strongly associated with the first time use of antidepressants [27]. A small number of studies have examined multimorbidity, breast cancer and depression in a less direct manner, principally through measurement of quality of life scores. Their findings generally support ours (while not being directly comparable), with Schoormans et al. reporting comorbidity to have a negative impact upon quality of life, and Deshpande et al. finding women with higher chronic disease burden have more depressive symptoms and higher levels of perceived stress [28, 29]. A minority of studies have also looked at the associations between specific co-morbidities and risk of depression among breast cancer survivors. One paper examining the influence of cardiovascular co-morbidity found no increased risk of depression amongst those with a comorbidity compare to without [15]. Further research outlining the individual associations between specific comorbid conditions and depression, as well as the potential mediating pathways (e.g. diet and physical activity) would be beneficial, especially as our descriptive results suggest that depression is higher among people with co-morbid diabetes and irritable bowel syndrome.

### Study limitations

Data from UK Biobank is not without flaws – one being evidence of a “healthy volunteer” selection bias. The average UK Biobank participant is healthier than the average member of the UK population. Additionally, participants in UK Biobank were in the age range of 40–70 at the time of recruitment, meaning the ages outwith this bracket are not represented. However, assessment of exposure-disease relationships, such as in the primary aim of this study, may remain generalisable without requiring participants to be reflective of the larger population [30].

Use of UK Biobank data enabled a large sample size of 8438 participants with breast cancer to be obtained and the inclusion of a range of potential confounding factors, the central strength of this study. A key limitation of this study is reliance on self-reporting of the exposure and outcome variables, which can be subjective and therefore less reliable than objective measures such as a formal diagnosis from medical records. However, as previously mentioned, the PHQ-2 is a valid tool for measuring depression and it enabled assessment of people with depression who may not have presented to health services [18]. We used the higher cut-off threshold of ≥3 to identify more severe depression and there are some potential limitations with this approach, including lower sensitivity, but higher specificity [19]. It is possible that using other tools to measure current depression would have produced different results and further work using other outcome measures is needed [31]. Our measure of multimorbidity was also based on a count of self-reported lifetime chronic conditions which may be an overestimate of those conditions currently experienced. However, it may be the case that using a measure of current multimorbidity would be more strongly associated with current depression. Further research using different definitions of multimorbidity is needed, including using administrative data from different sources (e.g. primary and secondary care).

A comparison to people without breast cancer would have been beneficial to this study, as would a comparison to people with other cancers. As a smaller subset of the breast cancer population, men are underrepresented in this area of research, and regretfully were not included in our analysis. Finally, the exclusion of participants with missing data has a risk of introducing bias, but only a small proportion of people were excluded for this reason (*N* = 926, 9.9%).

## Conclusions

This study finds clear evidence of an association between multimorbidity and depression among breast cancer survivors in UK Biobank. A dose-response relationship was observed in which the risk of depression increased alongside the increased number of comorbid conditions experienced. These results are of importance at this current time, when breast cancer patients are surviving for greater periods of time and thus experiencing the impact of multimorbidity to a greater extent. Increased awareness and effort to prevent depression and multimorbidity in cancer survivors is needed as they both contribute to decreased adherence to treatment, decreased cancer survival, increased suicidal behaviour, and greater health expenditure [32–34].

## Supplementary Information

**Additional file 1: Table S1.** Self-reported chronic health conditions included in the multimorbidity count.

## Data Availability

The data that support the findings of this study are available from UK Biobank (https://www.ukbiobank.ac.uk/), but restrictions apply to their availability. These data were used under licence for the current study, and so are not publicly available. The data are available from the authors upon reasonable request and with permission of UK Biobank.

## Abbreviations

CI: Confidence interval
IBS: Irritable bowel syndrome
ICD: International Classification of Diseases
OR: Odds ratio
N: Number of participants
PHQ: Patient Health Questionnaire
PHQ-2: Patient Health Questionnaire 2 item version
UK: United Kingdom
