# Supplementary material for: Associations between multimorbidity and depression among breast cancer survivors within the UK Biobank cohort: a cross-sectional study
Source: BMC Cancer. 2021 May 31;21:650. doi: 10.1186/s12885-021-08409-z (PMC8167936; doi:10.1186/s12885-021-08409-z)
Supplement: Supplementary file 1 — Additional file 1: Table S1. Self-reported chronic health conditions included in the multimorbidity count. [file 12885_2021_8409_MOESM1_ESM.docx]

**Table S1: Self-reported chronic health conditions included in the multimorbidity count (Adapted from Jani et al, 2019)**

Conditions highlighted in yellow were excluded from the count. UK Biobank data fields 20001 20002.

| **Chronic health condition group** | **Conditions included as reported by participants** |
| --- | --- |
| 1. Painful conditions | Back pain  Joint pain  Back pain  Joint pain  Headaches (not migraine)  Sciatica  Plantar fasciitis  Carpal tunnel syndrome  Fibromyalgia  Arthritis  Shingles  Disc problem  Prolapsed disc/slipped disc  Spine arthritis/spondylitis  Ankylosing spondylitis  Back problem  Osteoarthritis  Gout  Cervical spondylosis  Trigeminal neuralgia  Disc degeneration  Trapped nerve/compressed nerve |
| 1. Hypertension | Hypertension  Essential Hypertension |
| 1. Depression | Depression  Postnatal Depression |
| 1. Asthma | Asthma |
| 1. Atrial Fibrillation | Atrial Fibrillation |
| 1. Coronary Heart Disease | Heart attack/Myocardial Infarction  Angina |
| 1. Dyspepsia | Gastro-oesophageal reflux (GORD)/gastric reflux  Oesophagitis /Barrett's oesophagus  Gastric stomach ulcers  Gastric erosions/gastritis  Duodenal ulcer  Dyspepsia/indigestion  Hiatus hernia  Helicobacter pylori |
| 1. Diabetes | Diabetic nephropathy  Diabetic neuropathy/ulcers  Diabetes  Type 1 diabetes  Type 2 diabetes  Diabetic eye disease |
| \| 1. Thyroid disorders \| \| --- \| | Thyroid problem (not cancer)  Hyperthyroidism/thyrotoxicosis  Hypothyroidism/myxoedema  Grave’s disease  Thyroid goitre  Thyroiditis |
| 1. Connective tissue disorders | Myositis/myopathy  Systemic Lupus Erythematosus  Connective tissue disorder  Sjogrens syndrome/sicca syndrome  Dermatopolymyositis  Scleroderma/systemic sclerosis  Rheumatoid arthritis  Psoriatic arthropathy  Dermatomyositis  Polymyositis  Polymyalgia Rheumatica  Malabsorption/coeliac disease |
| 1. Chronic Obstructive Pulmonary Disease (COPD) | COPD/chronic obstructive airways disease  Emphysema/chronic bronchitis  Emphysema |
| 1. Anxiety | Anxiety/panic attacks  Nervous breakdown  Post-traumatic stress disorder  Obsessive compulsive disorder  Stress  Insomnia  Psychological/psychiatric problem |
| 1. Irritable bowel syndrome | Irritable bowel syndrome |
| 1. Alcohol problems | Alcohol dependency  Alcoholic liver disease/alcoholic cirrhosis |
| 1. Other psychoactive substance abuse | Opioid dependency  Other substance abuse/dependency |
| 1. Treated constipation | Constipation |
| 1. Stroke/Transient Ischaemic Attack (TIA) | Stroke  TIA  Subarachnoid haemorrhage  Brain haemorrhage  Ischaemic stroke |
| 1. Chronic kidney disease | Polycystic kidney  Diabetic nephropathy  Renal/kidney failure  Renal failure requiring dialysis  Renal failure not requiring dialysis  Kidney nephropathy  Immunoglobulin A (IgA) nephropathy |
| 1. Diverticular disease | Diverticular disease  Diverticulitis |
| 1. Peripheral vascular disease | Peripheral vascular disease  Leg claudication/intermittent claudication |
| 1. Heart failure | Cardiomyopathy  Hypertrophic cardiomyopathy  Heart failure/pulmonary oedema |
| 1. Prostate disorders | Prostate problem (not cancer)  Enlarged prostate  Benign prostatic hypertrophy |
| 1. Glaucoma | Glaucoma |
| 1. Epilepsy | Epilepsy |
| 1. Dementia | Dementia  Alzheimer’s disease  Cognitive impairment |
| 1. Schizophrenia/bipolar disorder | Schizophrenia  Mania/  Bipolar disorder  Manic depression |
| 1. Psoriasis/eczema | Eczema  Dermatitis  Psoriasis |
| 1. Inflammatory Bowel Disease | Inflammatory Bowel Disease  Crohn’s disease  Ulcerative colitis |
| 1. Migraine | Migraine |
| 1. Chronic sinusitis | Chronic sinusitis |
| 1. Anorexia or bulimia | Anorexia  Bulimia  Other eating disorders |
| 1. Bronchiectasis | Bronchiectasis |
| 1. Parkinson’s disease | Parkinson’s disease |
| 1. Multiple Sclerosis | Multiple Sclerosis |
| 1. Viral Hepatitis | Infective/viral hepatitis  Hepatitis B  Hepatitis C  Hepatitis D  Hepatitis E |
| 1. Chronic Liver disease | Oesophageal varices  Non infective hepatitis  Liver failure/cirrhosis  Primary biliary cirrhosis |
| 1. Osteoporosis | Osteoporosis |
| 1. Chronic fatigue syndrome | Chronic fatigue syndrome |
| 1. Endometriosis | Endometriosis |
| 1. Meniere’s disease | Meniere’s disease |
| 1. Pernicious Anaemia | Pernicious Anaemia |
| 1. Polycystic ovary | Polycystic ovary |
| 1. Cancer | Lifetime diagnosis |

Reference: Jani, B.D., Hanlon, P., Nicholl, B.I. et al. Relationship between multimorbidity, demographic factors and mortality: findings from the UK Biobank cohort. BMC Med 17, 74 (2019). https://doi.org/10.1186/s12916-019-1305-x
